# Real-Time Detection for Wheat Head Applying Deep Neural Network

**DOI:** 10.3390/s21010191

**Published:** 2020-12-30

**Authors:** Bo Gong, Daji Ergu, Ying Cai, Bo Ma

**Affiliations:** Key Laboratory of Electronic and Information Engineering (Southwest Minzu University), State Ethnic Affairs Commission, Chengdu 610041, China; gongbo1120@163.com (B.G.); 21500121@swun.edu.cn (Y.C.); martbox@163.com (B.M.)

**Keywords:** deep learning, wheat head, real-time object detection, SPP

## Abstract

Wheat head detection can estimate various wheat traits, such as density, health, and the presence of wheat head. However, traditional detection methods have a huge array of problems, including low efficiency, strong subjectivity, and poor accuracy. In this paper, a method of wheat-head detection based on a deep neural network is proposed to enhance the speed and accuracy of detection. The YOLOv4 is taken as the basic network. The backbone part in the basic network is enhanced by adding dual spatial pyramid pooling (SPP) networks to improve the ability of feature learning and increase the receptive field of the convolutional network. Multilevel features are obtained by a multipath neck part using a top-down to bottom-up strategy. Finally, YOLOv3′s head structures are used to predict the boxes of wheat heads. For training images, some data augmentation technologies are used. The experimental results demonstrate that the proposed method has a significant advantage in accuracy and speed. The mean average precision of our method is 94.5%, and the detection speed is 71 FPS that can achieve the effect of real-time detection.

## 1. Introduction

Wheat is one of the most planted grains in the world, almost all of which is produced for consumption and has high nutritional value. The Food and Agriculture Organization of the United Nations shows that global wheat production was 765.76 million tons in year 2019 [1]. These data suggest that the development of the global wheat industry is crucial to global food security and directly affects social stability. Breeding is particularly important to ensure a stable wheat yield. Predicting the yield of wheat is a key step in the breeding work. The traditional counting methods rely on manual observation, which is too subjective and has obvious defects. According to the results made by Madec et al. [2], the measurement error is about 10%.

At present, image-processing technology and shallow learning are mainly used to detect wheat head. For shallow-learning methods, Support-Vector Machines (SVMs), boosting, and logistic regression are supposed to represent the shallow-learning methods with one hidden layer node or without. Using the color, texture, shape, and other characteristics of the wheat head itself, an image classifier is constructed to complete automatic detection. Yangjun Zhu et al. [3] proposed a coarse-to-fine wheat-head detection method with two steps. A complex method [4] is used to classify the wheat head and the image background by binarization thresholds. K et al. [5] proposed an approach to detect the wheat-spike characteristics by utilizing the geometric properties with around 80% accuracy. The above types of detection methods rely on a large number of artificial feature designs and require certain experience. The detection accuracy is easily affected by noises such as light, angle, leaf color, and soil. Therefore, the detection accuracy needs to be improved.

Deep learning emphasizes the structure of the model with more hidden layers and highlights the importance of feature learning [6]. With the development of deep-learning theory and the improvement of hardware performance, deep learning has become the most advanced method of computer vision. Now there are a variety of popular computer vision tasks, such as object detection, instance segmentation, and semantic segmentation, all solved by deep neural networks. As well, the task of wheat-head detection can be solved by using deep neural networks because these methods greatly improve the efficiency of counting, reduce manual participation, and assist in the estimation of wheat yield.

In addition, these models have a high generalization ability, which reduces the pre-processing work of images and the dependence on experience. Deep neural networks can well promote the intelligent development of agricultural production. Zhao Zhang et al. [7] proposed a tool for wheat-lodging detection that was novel and effective, using GoogleNet and UAS RGB imagery. Pound et al. [8] applied CNN models to locate wheat spikes and spikelets in glasshouse condition. Buzzy et al. [9] used the convolutional neural networks and the Arabidopsis plant-images datasets to count the plant leaf. Based on the GAN and CNN, Ze Luo et al. [10] proposed a detector for pine cone. Hasan et al. [11] used the R-CNN network for training, and the average accuracy of wheat-ear detection was 93.4%. Spike counting from images is also by using a deep-learning method [12]. Although the use of deep-learning technology has obtained good performance, there are still serious problems. There is always a problem of tradeoff between the detection speed and the detection accuracy. The current methods for wheat-head detection still have this disadvantage. In terms of dataset there are also some problems, for example, insufficient datasets, and not taking into account the type of wheat, region, growth period, etc.

**Figure 1:**
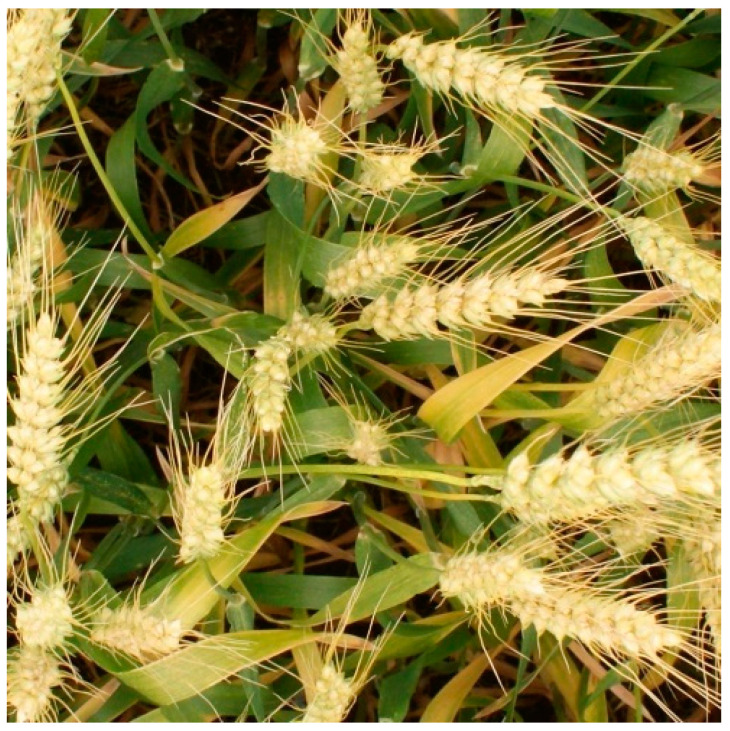
Example of an image in the wheat head dataset.

The main objectives of this paper are to detect the wheat head in the field images based on a deep neural network, and enhance the accuracy and the speed of the wheat-head detector. Specifically, we follow the latest development technology in the field of deep neural networks, and propose a novel method for wheat-head detection based on the object-detection algorithm YOLOv4 [13]. The backbone network is improved, and dual SPP is added to increase the receptive field. Meanwhile, the CSPNet is used to intergrade the multilevel features. In addition, the latest global wheat-head datasets GWHD [14] are employed to train the proposed method, as shown in Figure 1. The proposed method can detect wheat heads quickly and accurately, and also has a good ability of generalization.

The rest of the paper is organized as follows. Section 2 briefly reviews the related work. The proposed method is illustrated in Section 3. The experiments are conducted in Section 4. The results are discussed in Section 5 and concluded in Section 6.

## 2. Related Work

Two-stage object detection. As the basic task of computer vision, object detection has developed a series of excellent deep-learning models. The current object-detection frameworks are mainly divided into two types: single-stage and two-stage. The representatives of two-stage detectors include R-CNN [15], Fast R-CNN [16], Faster R-CNN [17], FCOS [18], etc. R-CNN applies deep learning to the field of object detection, laying a foundation for two-stage target detection. In the selection of region proposals, R-CNN uses the selective-search algorithm [19]. In the classification stage, the Support-Vector Machines (SVM) algorithm is applied. Girshick et al. [16] proposed the Fast R-CNN on the basis of R-CNN, and its innovation was that it eliminated the need to send all candidate boxes into the convolutional neural network. However, this method only needed to send one picture to the network. According to the previous experience, Ren et al. [17] proposed a two-stage object-detection method, Faster R-CNN, with faster detection speed. This method introduces the region proposal network (RPN), which extracts candidate-bound boxes by setting anchor boxes with different proportions and realizes an end-to-end network.

One-stage object detection. The representatives of single-stage object detection methods include Yolo series models (YOLO [20], YOLO9000 [21], YOLOv3 [22], YOLOv4 [13]) and SSD. One advantage of the single-stage target detection algorithm is a fast detection speed. For example, the detection speed of YOLO can reach 45 FPS. The idea of Yolo is to divide the input image into an S × S grid, and the grid generates a certain number of bounding boxes when the center of an object falls into it. Finally, frame and classify the objects by using non-maximum suppression (NMS) to select the appropriate prediction bounding boxes. According to the previous research of Yolo, Redmon et al. [21] proposed a novel method, YOLO9000. The main innovation of YOLO9000 is the use of multiple computer vision techniques, such as batch normalization, high-resolution classifier, location prediction, etc. The detection accuracy of YOLO9000 is 78.6% on the VOC2007 dataset. The backbone of YOLO9000 is Darknet-19, with a 3 × 3 convolutional kernel and global average pooling, which reduces the computational complexity and parameters of the model. Redmon et al. [22] put forward YOLOv3, which used the Darknet-53 network as the backbone network, and introduced the FPN [23] network to achieve the purpose of multiscale integration.

**Figure 2:**
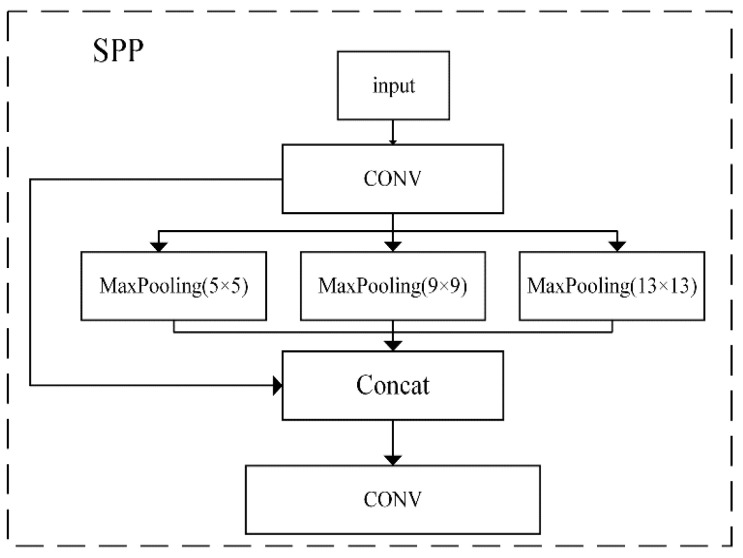
The structure of the spatial pyramid pooling (SPP) network with three different types of max pooling.

**Figure 3:**
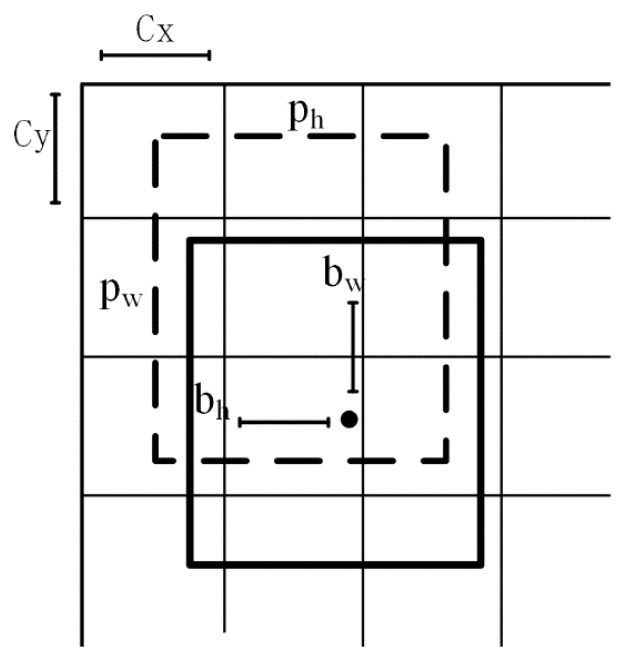
The bounding boxes of prediction and ground truth. p_h_ and p_w_ are the width and height of the ground-truth bounding, respectively. b_w_ and b_h_ are the bounding boxes of prediction, respectively.

Components of YOLOv4. YOLOv4 [13] was proposed by Alexey et al. The original intention of YOLOv4 was to optimize parallel computing and improve the speed of object detection. A deep neural-network object detector is composed of three parts: backbones, neck, and heads. The function of each part is different. The backbone part mainly extracts the features. The neck is used to fuse the features extracted from the main part. The role of the head is to predict, including predicting the bounding boxes and the object classification. The backbone network part of YOLOv4 applies CSPDarknet53 [24]. The CSPDarknet53 network is a Cross Stage Partial Network (CSPNet) added on the basis of Darknet53 [22]. Darknet53 draws on the idea of ResNet [25] to ensure that the network has depth while also alleviating the vanishing-gradient problem. CSPNet can enhance the learning ability of CNN while reducing the amount of calculation and memory cost. A good detector should have a larger receptive field. The neck of the YOLOv4 network uses two networks, SPP [26] and Path Aggregation Network (PAN) [27]. The SPP network applied in the neck can effectively increase the receptive field and help separate contextual features, as shown in Figure 2. The PAN plays a role in shortening the path connecting low-level information and high-level information, and converging parameters at different levels. The YOLOv4 network head inherits the head structure of YOLOv3. The head predicts the bounding box of the object, and outputs the center coordinates, width, and height, i.e., {x_center_, y_center_, w, h}. Then the expression of the predicted bounding box is shown as follows.

(1) $$\begin{array}{c} b_{x}=\sigma \left(t_{x}\right){+c}_{x}\\ b_{y}=\;\sigma \left(t_{y}\right){+c}_{y}\\ b_{w}{=p}_{w}\cdot e^{t_{w}}\\ b_{h}{=p}_{h}\cdot e^{t_{h}} \end{array}$$

where P_w_ and P_h_ represent the width and height of the prior bounding box, respectively. Also shown, (c_x_, c_y_) is the coordinate of the top left corner of the image. Each bounding box can be described by t_x_, t_y_, t_w_, and t_h_. Figure 3 shows the size of the prior bounding box and the position of the predicted bounding box.

## 3. Methods

**Figure 4:**
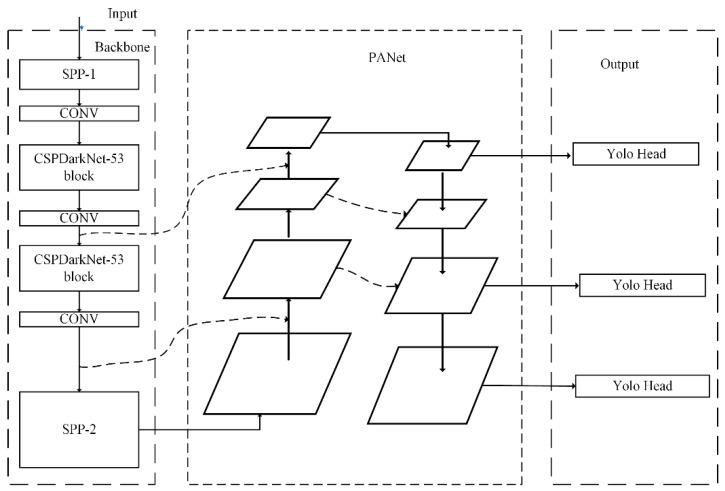
The structure of our proposed method consists of three parts. Two SPPs are added to the backbone part, and multipath is the feature of the neck part. For the last one, the head of our proposed method inherits from YOLOv3.

In this paper, the proposed method with real-time and accurate features is illustrated in Figure 4, which is mainly composed of three parts: backbones, neck, and heads. For the backbone, the SPP is applied to improve the receptive field of the network. Owing to the big size of the image in this article—1024 × 1024—the use of scaling and cropping operations will make the picture with more noise. To solve this problem, we added a spatial pyramid pooling network to the front of the backbone network. In addition, a fixed-size feature vector and the original image information are output effectively. The SPP can solve the multi size problem of the input image through multiscale pooling.

### 3.1. Dual SPP on the Backbone of Our Detector

**Figure 5:**
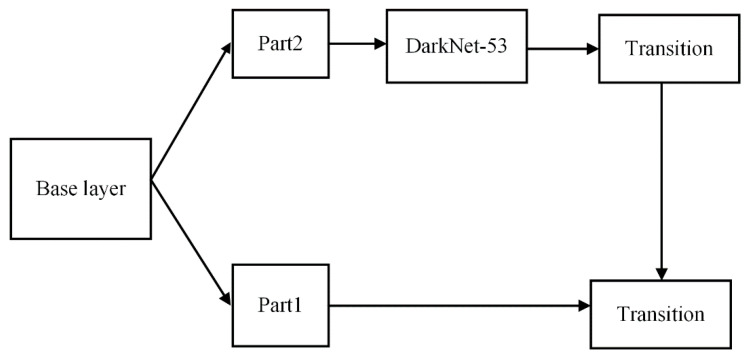
The structure of CSPDarkNet53 network. This is one kind of feature fusion strategies, transition→concatenation→transition.

An excellent backbone network should learn as many features of pictures as possible. To enhance the learning ability of the backbone network, a Cross Stage Partial Network (CSPNet) [24] is applied. CSPNet was proposed by Wang et al. to enhance the learning ability of convolutional neural networks. This network can still maintain or enhance the learning ability of the CNN network while reducing the amount of calculation by 20%. Therefore, in order to enhance the learning ability of the backbone network, we use the CSPNet network and obtain a new network structure, CSPDarkNet53 [24]. The structure is shown in Figure 5 and consists of two parts: skip connection and main part. The main part retains the original DarkNet53 structure, and multiple residual blocks are stacked. The skip-connection part is directly connected to a concat layer of the network, and it is also spliced with the main part.

In this paper, SPP [26] is introduced to the tail of the backbone network, denoted as SPP-2. In addition, the purpose of introducing SPP-2 is different from SPP-1. This part is to separate the context features and facilitate the neck network fusing the global feature information. Firstly, the SPP-2 network performs a convolution operation on the input features of the upper layer. Then, it performs a maximum pooling operation of different scales. The pooling size of pool-1, pool-2, and pool-3 is 5, 7, and 13, respectively. In addition, the step size is 4, 6, and 13, respectively. SPP-2 merges the output features of the three pooling layers and inputs them to the next convolutional module to perform feature learning to obtain the abundant local features.

### 3.2. MultiPath Neck

The low-level information contains the outline of the object. On the contrary, the high-level features contain the details of the target. In the field of object detection, the part of the detector that collects feature maps is usually called the neck. The neck usually consists of a bottom-up path and a top-down path. In view of the particularity of the task in this paper, the photos of wheat heads contain obvious structural features and abundant detailed features. We use PAN as the neck of the detector to collect multilevel features and connect with the SPP-2 to form a bottom-up and top-down combination. The inputs of the neck of our proposed method come from three parts, two of which are from the feature layer of the backbone network, and the other input is from the feature layer of SPP-2. The output of the path-aggregation network is used as the input for the head of the object-detection network.

The model in this paper uses YOLOv3 as the head to predict the bounding box. First, calculate the coordinates, width, and height of the prediction boxes according to Formula (1). Secondly, the confidence threshold is set to filter out the prediction frames with low scores. Finally, non-maximum value suppression is used to determine the final prediction frame.

### 3.3. Loss Function

**Figure 6:**
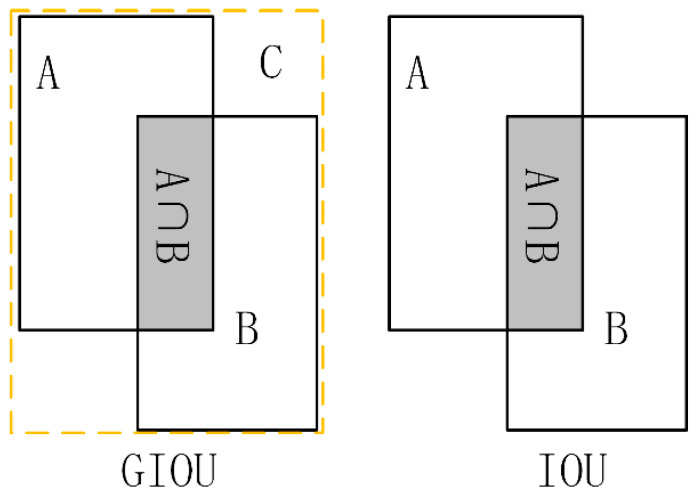
GIOU, the loss function used in our method. The comparison of GIOU and IOU makes obvious that GIOU is feasible to optimize with non-overlapping bounding boxes, but IOU is not.

The current object-detection method uses IOU to determine the degree of overlap between the predicted and the ground-truth bounding box. IOU is represented as:

(2) $$\mathrm{IOU}\;=\;\frac{M\;\cap \;N}{M\;\cup \;N}$$

where M is the prediction bounding box and N is the ground-truth bounding box. However, with this optimization method IOU has the disadvantage of not being able to optimize non-overlapping parts. Therefore, we introduce the generalized GIOU [28] loss function, represented as:

(3) $$\mathrm{GIOU}\;=\;\mathrm{IOU}-\frac{\left|A_{c}-U\right|}{\left|A_{c}\right|}$$

where A_c_ represents the minimum bounding box between the predicted bounding box and the ground-truth bounding box. U is the union of the predicted and the ground-truth bounding boxes, i.e., M∪N. The loss function not only pays attention to the overlapping area but also focuses on the non-overlapping area of the two kinds of boxes, which better reflects the overlap of the two boxes. Shown in Figure 6 are the intuitive schematic diagrams of IOU and GIOU, respectively. The bounding-box regression-loss function used in this article is:

(4) $${\mathrm{Loss}}_{\mathrm{GIOU}}\;=\;1\;-\mathrm{GIOU}$$

The value range of GIOU is (−1, 1). The higher the overlap of the bounding box M and N, the closer the GIOU is to 1. When M and N do not overlap, optimization can still be performed, which benefits from the existence of the smallest bounding boxes. By contrast, this advantage is missing from IOU.

## 4. Experiments

### 4.1. Datasets

**Figure 7:**
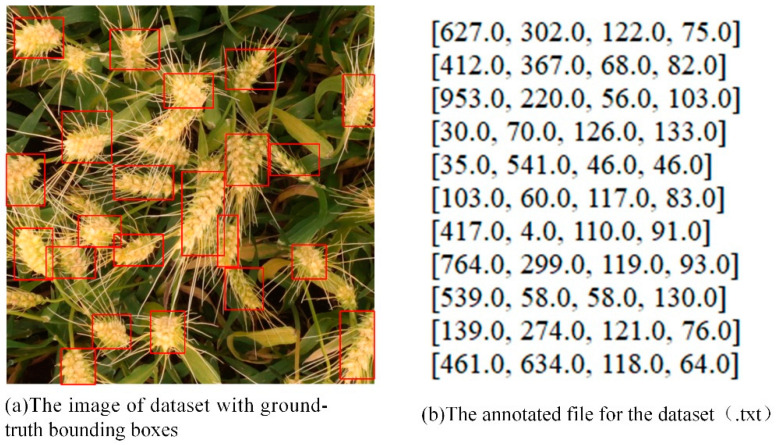
An example of dataset annotation. (**a**) is the vision of ground-truth bounding boxes. (**b**) is the txt format file including the four parameters of each wheat-head label.

The dataset applied in this paper is the Global Wheat Head Detection dataset GWHD [14]. The GWHD dataset was constructed collaboratively by numerous countries. The GWHD dataset is the first large-scale dataset to detect wheat heads from field optical images. The wheat-head pictures are varieties grown in different regions. The dataset uses the web-based annotation tool coco annotator [29]. The platform is rich in features, with all the tools required to label objects. Labeling the large-density bounding boxes is difficult. Therefore, it is required to draw a box containing all the pixels of the wheat head when the image is complete or the part of the veins is occluded. The labeled part contains at least one wheatear. Figure 7 shows the ground-truth boxes and label files of part of the dataset. The label information is the top-left coordinates (x_min_, y_max_) of the bounding box and the width and height of the bounding box w, h. In consideration of the numerous GWHDs, some data augmentation techniques are referenced, especially online augmentation. Table 1 shows all the data augmentation techniques we used.

### 4.2. Evaluation Metrics

To evaluate the effect of wheat-head detection, the following evaluation metrics are used:

(1) Recall and precision:

(5) $$P_{\mathrm{recision}}=\frac{\mathrm{TP}}{\mathrm{TP}+\mathrm{FP}}$$

(6) $$R_{\mathrm{ecal}}=\;\frac{\mathrm{TP}}{\mathrm{TP}+\mathrm{FN}}$$

True positive (TP) represents that samples are predicted to be correct and actually positive. False-Positive (FP) represents that samples are predicted to be positive but negative actually. In addition, False-Negative (FN) represents that samples are predicted to be negative but positive actually.

(2) The AP is to average the precision, and it is shown in Equation (4):

(7) $$\mathrm{AP}=\;\int_{0}^{1}P(R)\mathrm{dR}$$

In practice, the PR curve is smoothed, and the area under the curve is used to calculate the object’s AP value.

(3) Mean Average Accuracy (mAP) means that the average accuracy of all categories is added, and it is divided by the number of categories, shown as follows:

(8) $$\mathrm{mAP}=\;\frac{\sum_{i=1}^{N}{\mathrm{AP}}_{i}}{N}$$

where N is the number of object classifications. We use two ranges of average precision mean, mAP_50_, and mAP_95_. The mAP_50_ represents the average accuracy of the confidence threshold of 50%, which is recorded as mAP_50_ in this article. The mAP_95_ represents the mean value of the average accuracy in the range of 50–95% confidence level, which is expressed directly in this article.

(4) Frame Per Second (FPS), means the number of images that can be detected per second, used to evaluate the speed of the detector. Only the detection speed of the method is fast enough to realize real-time detection and meet the needs of the industry.

### 4.3. Training

**Figure 8:**
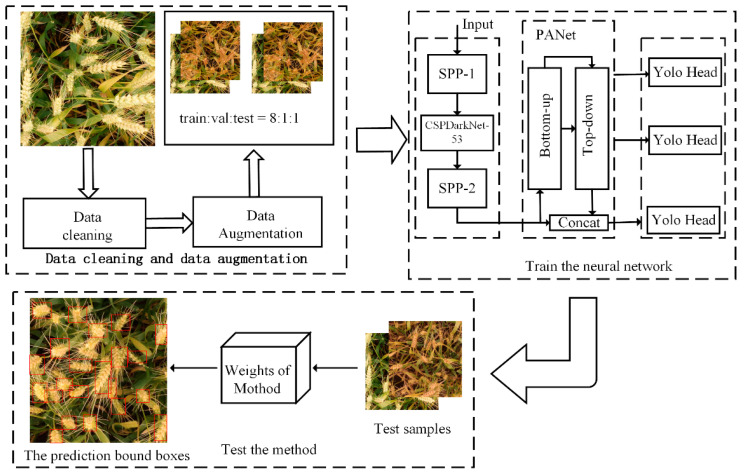
The work-flow of training and testing our proposed detector for wheat head. The three steps are data augmentation, training network, and testing the method.

In our experiment, we used Intel(R) Xeon(R)Silver 4110 CPU (Intel, Santa Clara, California, USA), and GPU is GeForce RTX 2080 Ti (NVIDIA, Santa Clara, California, USA) for accelerating model training. The programming language was python 3.7 in this paper, based on Pytorch 1.5. The specific process of this experiment is shown in Figure 8. The steps of this experiment were as follows: Firstly, we removed some pictures according to the size of the bounding box from the original dataset, keeping the dataset with accurate and clean labels. Then, a series of data enhancement operations were performed, including rotation, cropping, adding noise, etc. Finally, the training datasets were input into the deep neural network for training.

**Figure 9:**
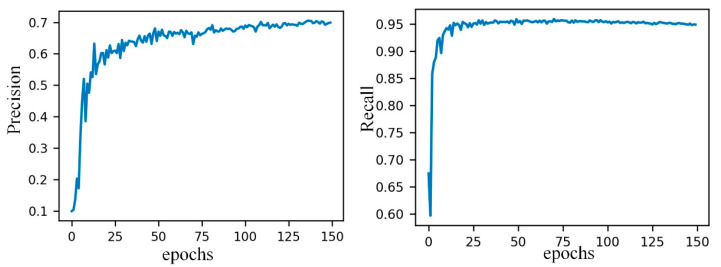
The curves of precision and recall about training.

In the training phase of this paper, all models were trained for 150 epochs, using SGD (stochastic gradient descent optimization), and momentum and decay weights were set as 0.937 and 0.0005, respectively. The batch size was 16, and the initial learning rate was 0.01. The training accuracy and recall-rate change curve are shown in Figure 9.

## 5. Results and Discussion

### 5.1. Wheat-Head Detection Results

**Figure 10:**
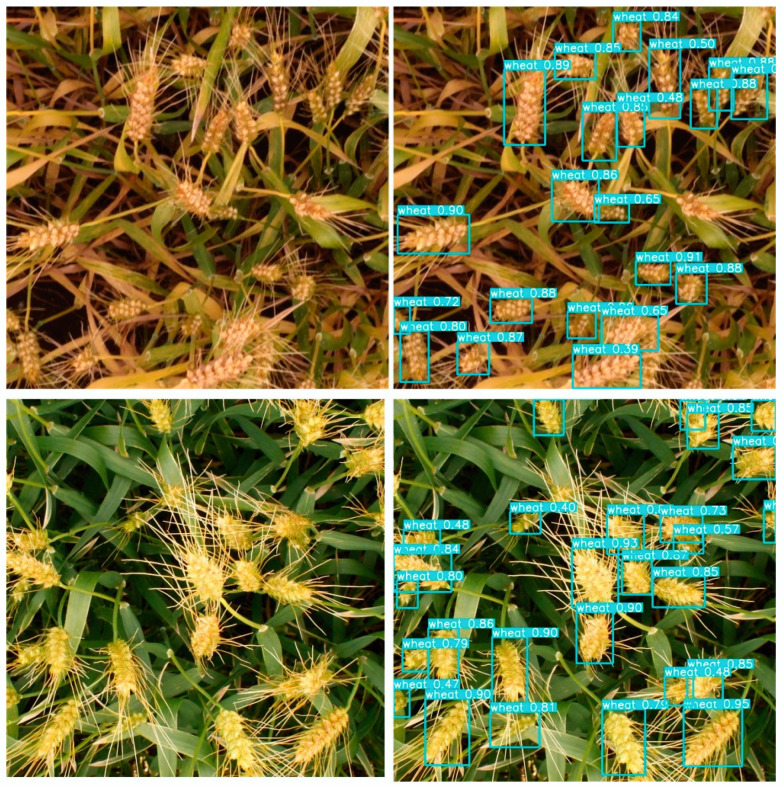
Example of wheat-head detection. The left images are the testing images without any label. The right images are the location prediction of wheat head with boxes.

It can be seen from Figure 10 that the proposed method performs well for wheat-head detection. The detection effect of the wheat head at the mature stage is better, which benefits from the characteristic integrity and uniqueness of the wheat head at the mature stage. In order to scientifically show the detection performance of our method, we trained and tested the proposed method with other detectors on the same dataset and compared the test results. Table 2 shows the detection performance of each detector.

It can be seen from Table 2 that our proposed method in this paper has achieved good results on the task of wheat-head detection. Compared with the YOLOv3, the mAP_50_ and mAP_95_ indexes of our method are improved by 4% and 7.8%, respectively. In addition, the detection speed also increases by 33 FPS. Compared to YOLOv4, our proposed method increases mAP_50_ and mAP_95_ up to 3.1% and 3.3%, respectively. The detection speed increases by 19 FPS. Compared with the two-stage detection method, Faster R-CNN, our method in this paper does not lose advantage in accuracy. Its mAP_50_ and mAP_95_ increase by 17.9% and *5*.4%, respectively. The speed of our detector increases by 61 FPS. In general, our proposed method in this paper achieves a good performance in detecting wheat heads, and its performance is slightly better than YOLOv4. Our method guarantees the speed and accuracy of the detector all have a good performance.

### 5.2. Comparison of Different Backbone Model Detection Indexes

In order to illustrate the influence of different backbones on our detector, we experimented and confirmed the three backbone networks. While keeping the neck and head of our method unchanged, DarkNet-53, CSPDarkNet-53, and the backbone of our proposed method were used for the experiment. Table 3 lists the wheat-head detector accuracy and the speed of the detector.

It can be seen from Table 3 that compared with the other two backbone networks, the improved backbone in this paper increases mAP_50_ by 6.9% and 4.3%, respectively. In addition, our proposed backbone improves mAP_95_ by 5.9% and 4.1%, respectively. In terms of detection speed, using the improved backbone is faster than the detector based on DarkNet-53 and CSPDarkNet-53 by 16 FPS and 6 FPS, respectively. After comprehensive consideration, our proposed backbone still has certain advantages that have the ability to enhance the accuracy and the speed of the wheat-head detector. As the result of the data augmentation, fewer labeled images are needed and the training time is reduced to only 22 h, less than the training time without data augmentation.

## 6. Conclusions

Wheat-head detection is a valuable method for wheat-production estimation, wheat breeders, and crop management. In order to enhance the performance of the wheat-head detector, in this paper a novel object method with dual SPP networks is proposed in the backbone part. In addition, a multipath neck component with a bottom-up to top-down strategy is proposed, which integrates effectively multiscale features. GIOU used as a loss function also largely reduces the training time. The experimental results show that the proposed method effectively solves the task of wheat-head detection and significantly outperforms other methods. The accuracy is 94.5%, and the detection speed is 71 FPS. However, there are some shortcomings of this study, e.g., we only tested one type of dataset in experiments, and the accuracy of the proposed method needs to be higher. Therefore, in future work we will consider updating our research and introduce a self-attention mechanism to enhance the feature-learning ability of the detector. Regarding the datasets, we consider to use a generative adversarial network for data augmentation and testing on other types of datasets.

## Figures and Tables

**Table 1:** Detailed information about wheat-head datasets. Some data augmentation techniques are applied to the original training datasets.

Data Augmentation	Training	Validation	Testing	Total
Original	3412	10	10	3432
Cutout	√	-	-	
Crop	√	-	-	
Blur	√	-	-	
Flips	√	-	-	

**Table 2:** The comparisons of numerous detection methods.

Method	Datasets	mAP_50_	mAP_95_	FPS
YOLOv3 [13]	GWHD	90.5%	46.7%	38
YOLOv4 [14]	-	91.4%	51.2%	52
Faster R-CNN [8]	-	76.6%	49.1%	10
Our method	-	**94.5%**	**54.5%**	**71**

**Table 3:** Using different backbones for wheat-head detector training.

Backbones	Datasets	mAP_50_	mAP_95_	FPS
DarkNet-53	GWHD	87.6%	48.6%	55
CSPDarkNet-53	-	90.2%	50.4%	65
Our backbone	-	94.5%	54.5%	71

## Data Availability

The data presented in this study are available on request from the corresponding author.
